# Research Progress on Anticancer Mechanism of Ginsenoside Regulating Tumor Microenvironment

**DOI:** 10.3390/cimb48030329

**Published:** 2026-03-20

**Authors:** Tianjia Liu, Wei Li, Da Liu, Baiji Xue

**Affiliations:** 1 College of Pharmacy, Baicheng Medical College, Baicheng 137000, China; ltj@bcmc.edu.cn; 2 College of Pharmacy, Changchun University of Chinese Medicine, Changchun 130117, China; lw17638310029@163.com; 3 Public Experimental Center, Changchun University of Chinese Medicine, Changchun 130117, China; 4 School of Basic Medicine, Baicheng Medical College, Baicheng 137000, China

**Keywords:** ginsenoside, tumor microenvironment, cancer, lipidosome, nano drug delivery system

## Abstract

Cancer is currently one of the most significant health threats facing humanity in general. The clinical treatment of cancer is constrained by the current development of chemotherapy drug resistance, poor pharmacokinetics, off-target toxicity, and insufficient intratumoral accumulation. Although surgery combined with chemotherapy is now maturely used in clinical practice, the results are unsatisfactory, and the incidence and mortality of cancer continue to increase year by year with high side effects from treatment. Therefore, it is important to find more effective therapeutic targets against cancer. Alterations in the tumor microenvironment can lead to cellular gene mutations, which are an important cause of tumorigenesis, and therapeutic interventions targeting the tumor microenvironment have been one of the most interesting research areas in the oncology community in recent years. Ginseng is rich in antitumor-active ingredients and is used in the treatment of many cancer diseases. Ginsenoside is one of the main active components of ginseng. This paper reviews the antitumor mechanism of action of ginsenoside through regulating the tumor microenvironment, emphasizing the key role of ginsenoside in the tumor microenvironment and providing a new target and theoretical basis for ginsenoside in the treatment of cancer.

## 1. Introduction

Ginseng is a very precious medicinal herb that grows in China, Russia and Korea, and is known as one of the “Three Treasures of the Northeast” in China [1,2]. Among them, ginsenosides are the main active components with various pharmacological effects, such as antitumor [3,4], anti-inflammatory [5,6], hypoglycemic [7,8], antidepressant [9], and immunomodulatory [10,11]. At present, in antitumor scientific research, ginsenosides and their metabolites are the hot spot and will be the most important adjuvant therapeutic agents for tumors [12]. More than 80 ginsenoside components have been isolated from ginseng [13], and these ginsenosides can inhibit the growth [14], invasion [15,16] and metastasis [17] of tumors. For example, ginsenosides have been widely used in therapeutic research on several types of tumors: lung cancer [18], prostate cancer [19], colorectal cancer [20], ovarian cancer [21], and breast cancer [22].

Among these, Rg3, Rh2, Rg1, Rb2, and CK exhibit the closest associations with the regulation of the tumor microenvironment (TME). The structural features of these monomers—encompassing aglycone types, glycosylation modifications, and isomeric forms—largely dictate their targeting selectivity and pharmacological activities. From the perspective of aglycones, protopanaxadiol (PPD)-type saponins (Rg3, Rh2, Rb2, CK) tend to exert effects on tumor cells or vascular endothelial cells, whereas protopanaxatriol (PPT)-type saponins (Rg1, Rh1) primarily modulate immune cells [23]. The length of sugar chains also influences their mode of action: the polysaccharide chain structures of Rb2 and Rg1 confer favorable water solubility, facilitating their interaction with fibroblasts or immune cells within the TME. In contrast, saponins with fewer or no sugar chains (e.g., Rg3, Rh2, CK) exhibit higher lipophilicity, enabling easier penetration of cell membranes and thus primarily targeting vascular endothelial cells [24,25]. Additionally, isomerism contributes to functional divergence. For instance, 20(S)-Rg3 displays stronger activity in anti-angiogenesis and the inhibition of epithelial–mesenchymal transition (EMT), while 20(R)-Rg3 demonstrates greater efficacy in regulating the immune microenvironment [26].

Cancer is one of the leading killers of human health and kills more than 5 million people worldwide each year [27,28]. China has 2.4 million new cancer cases and 1.3 million deaths each year [29]. With up to $20 billion spent annually on cancer treatment worldwide, cancer causes tremendous suffering to patients and their families and imposes a huge economic burden on society [30]. Therefore, there is an urgent need to develop new drugs for cancer treatment [31]. At present, the research of cancer therapy is no longer limited to the treatment of tumor cells because there are complex interactions between the tumor microenvironment and tumor cells in the process of tumor occurrence and development. This process has been summarized in some very important landmarks, including persistent proliferative signals [32], growth inhibition regulation [33], anti-apoptotic cell death [34], permanent replication capability [35], neovascularization [36], invasion and metastasis [37], energy metabolism reprogramming [38], evasion of immune attacks [39], obtaining phenotypic plasticity, etc. These signature features can guide subsequent cancer researchers in the search for the corresponding important molecular mechanisms for specific cancer subtypes and in the development of preventive and therapeutic approaches. Tumor microenvironment (TME) refers to the complex micro-ecological environment in which tumors occur and develop, which is a major feature in addition to the six major features of tumors [40]. The tumor microenvironment is composed of tumor cells, extracellular matrix, lymphocytes, vascular-associated endothelial cells, immune cells, fibroblasts, extracellular factors, chemokines, etc. These components are interrelated and interact with each other and play a role in the development of tumors [41]. Notably, many of the hallmark features of tumors demonstrate the important role of the “tumor microenvironment” [42]. In other words, a tumor is not just a collection of relatively homogeneous cancer cells but a complex system of tissues in which the cancer cells themselves are highly heterogeneous due to genetic mutations and phenotypic plasticity, while the cancer cells recruit many normal cells that have not undergone the original oncogenic mutations into the tumor tissue and modify them, thus undergoing complex interactions. The tumor microenvironment significantly influences the biological behavior of cancer cells and their response to therapeutic approaches in many important ways [43]. A variety of drugs targeting tumor microenvironment therapy have been widely used in clinical practice, such as drugs targeting tumor angiogenesis that promote vascular normalization by blocking angiogenesis of endothelial cells at the tumor site. Therefore, targeting the tumor microenvironment is a promising therapeutic approach for tumor treatment. This paper summarized the research progress of ginsenoside regulation of the tumor microenvironment for tumor treatment, laying a foundation for clinical treatment.

This review aims to provide a comprehensive overview of the regulatory effects of ginsenosides on the tumor microenvironment (TME) and their implications for cancer therapy. The scope of this review encompasses three key aspects of TME modulation—tumor angiogenesis, epithelial–mesenchymal transition (EMT), and immunosuppression—with particular emphasis on the structural diversity of ginsenosides (PPD-type vs. PPT-type, glycosylation patterns, and stereoisomers) as determinants of their differential targeting selectivity. Unlike previous reviews that focus on individual ginsenosides or mechanisms, this work systematically integrates evidence from in vitro, in vivo, and clinical studies to elucidate how specific structural features dictate the interaction of ginsenosides with distinct TME components. The originality of this review lies in its structure–function perspective, providing a framework for understanding how ginsenoside monomers selectively target vascular endothelial cells, immune cells, or tumor cells based on their aglycone type and sugar chain modifications, thereby offering new insights for the rational design of ginsenoside-based combination therapies and targeted delivery systems.

## 2. Ginsenoside Regulates Tumor Angiogenesis in the TME of Cancer

Angiogenesis, the formation of new blood vessels from existing ones, is a key process in the proliferation, migration and differentiation of cancer cells. During the development of malignant tumors, tumor angiogenesis plays an important role in the rapid growth and metastasis of tumors when tumor neovascularization is out of control [44,45]. Therefore, targeting angiogenesis is an effective method of treating tumors [46]. A number of anti-angiogenic targeted agents are now being used in clinical practice as various oncology treatment strategies and to improve clinical patient survival outcomes. Anti-angiogenic therapy has been successfully applied in the treatment of colorectal cancer.

### 2.1. In Vitro Evidence of Anti-Angiogenic Effects

Ginsenoside Rg3 (GS-Rg3) has an anti-angiogenic effect and inhibits tumor growth and metastasis [47]. Studies have shown that GS-Rg3 targets tumor stem cells and tumor angiogenesis in vivo to inhibit colorectal cancer progression. The results showed that GS-Rg3 can downregulate the expression of angiogenesis-related genes and inhibit xenograft vascularization in colorectal cancer cells, and that Rg3 reshapes the tumor microenvironment by inhibiting tumor angiogenesis and promoting antitumor immunity [48].

VEGF plays an important role in angiogenesis. VEGFR2 is a receptor tyrosine kinase (RTK), one of the three isoforms of the VEGF receptor. The interaction between VEGF and VEGFR2 is thought to be a key driver of angiogenesis [49]. One of the common mechanisms to inhibit angiogenesis is to reduce the expression of VEGF and VEGFR2. In a study evaluating the efficacy of hepatic arterial administration of GS-Rg3 in combination with transcatheter arterial embolization (TAE) for the treatment of liver tumors, immunohistochemical staining, semi-quantitative RT-PCR, and Western blotting were used to detect the expression of the angiogenic biomarkers CD31 and VEGF and the apoptotic markers caspase-3, Bax and Bcl-2. These results demonstrate that GS-Rg3 significantly inhibits HepG2 cell proliferation, downregulates VEGF expression, inhibits tumor angiogenesis and induces apoptosis to effectively inhibit tumor growth in an in vitro assay [50].

Tumor metastasis is one of the key factors in thyroid cancer becoming a high-risk cancer. Studies have demonstrated that GS-Rg3 significantly inhibits metastasis of thyroid cancer, including four papillary thyroid carcinoma (PTC) cells (TPC-1, BCPAP, C643, oct -2c), both in vivo and in vitro, and inhibits lung metastasis of C643 cells in a nude mouse lung metastasis model. GS-Rg3 was found to reduce the expression levels of matrix metalloproteinase-2 (MMP-2) and MMP-9 proteins in four types of thyroid cancer cells and to inhibit the expression of vascular endothelial growth factor-c (VEGF-C) in PTC cells and VEGF-A protein in interstitial thyroid cancer (ATC) cells, which suggests that GS-Rg3 may inhibit PTC lymph node metastasis and angiogenesis in ATC [51].

Ginsenoside Rh2 (GS-Rh2) was found to inhibit angiogenesis in prostate cancer by targeting CNNM1.

LNCaP, PC3 and DU145 were co-cultured with vascular endothelial cells, and the expression levels of CD31, VEGF, PDGF and CNNM1 were detected by qRT-PCR and Western blot. The results showed that GS-Rh2 inhibited the development of prostate cancer by reducing the expression of CNNM1 in cancer cells to suppress their angiogenesis [52].

A study on ginsenoside Rg1 (GS-Rg1)-induced MDA-MB-MD-231 apoptosis in triple-negative breast cancer cells confirmed that GS-Rg1 downregulated invasion and angiogenic markers and inhibited cell proliferation. The authors found that GS-Rg1 induced cytotoxicity and apoptotic cell death by generating reactive oxygen species (ROS) and altering mitochondrial membrane potential (MMP) in triple-negative breast cancer cells through in vitro and in vivo experimental models [53].

**Figure 1:**
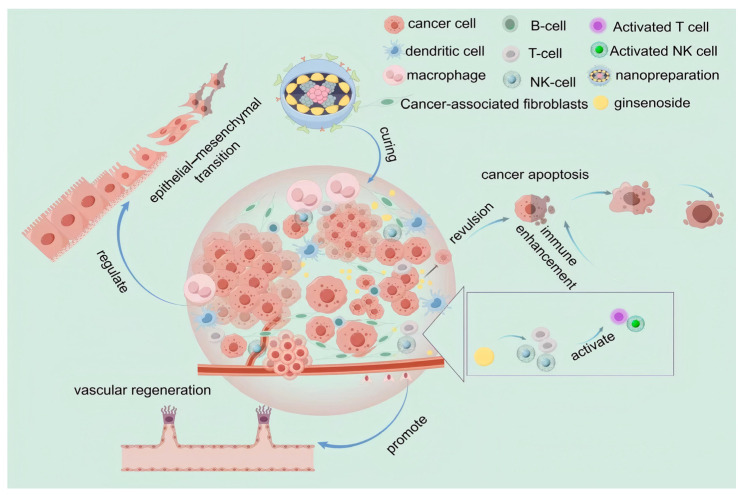
Ginsenosides decrease tumor angiogenesis and inhibit tumor development. Created in BioRender. Li, W. (2026) https://BioRender.com/337kd3w.

The key ginsenosides, their target genes, and associated cancer types are summarized in Table 1, and the major signaling pathways involved in angiogenesis regulation are illustrated in Figure 1.

### 2.2. In Vivo Evidence from Animal Models

It has been found that GS-Rg3 sensitizes colorectal cancer to radiation in vivo. CT-26 xenograft tumors were established in BALB/c mice and treated with drug-loaded, Rg3, radiotherapy and Rg3 + combined radiotherapy to demonstrate that Rg3 + combined radiotherapy can effectively inhibit angiogenesis, thereby suppressing tumors and prolonging the life span of CT-26 xenograft BALB/c mice [64].

Notably, the combination of GS-Rg3 with chemotherapeutic agents has demonstrated synergistic effects. Research indicates that in colon cancer-bearing mice treated with Rg3 combined with 5-fluorouracil (5-FU), the tumor inhibition rate in the combination group reached 73.42%, with a tumor necrosis rate of 55.63%, significantly higher than that of the 5-FU monotherapy group (68.78% inhibition rate). The combination treatment also reduced tumor vascularization (CD31+ microvessel density and VEGF expression) and improved survival, with 70% of mice surviving at day 42 compared to 50% in the 5-FU group [54].

Meng et al. found that Rg3 downregulated VEGF in melanoma cells and inhibited endothelial cell proliferation and migration, thereby suppressing melanoma-induced angiogenesis [61]. It was shown that GS-Rg3 induced apoptosis in hepatocellular carcinoma (HCC) cells in vitro, and Nakhjavani et al. further explored the cross-linked effects of GS-Rg3 in inducing apoptosis and anti-angiogenesis in vivo. GS-Rg3 was orally administered to mouse liver cancer cells after transplantation into the liver to analyze apoptosis, and the results showed that Rg3 induced apoptosis and inhibited angiogenesis. GS-Rg3 initiated the apoptotic process in the tumor, which in turn weakened the tumor volume and its ability to generate a vascularized network for further tumor growth and distant metastasis [55]. In rats with gastric precancerous lesions, CD34+ microvessel density and VEGF expression were significantly increased, and administration of GS-Rg3 reduced VEGF protein expression and CD34+ microvessel density. Meanwhile, data analysis showed that GLUT1, GLUT3 and GLUT4 protein expression was enhanced in human and animal rats with gastric precancerous lesions; thus, it can be demonstrated that GS-Rg3 attenuated angiogenesis and moderated microvascular abnormalities in rats with gastric precancerous lesions, which may be related to GS-Rg3 inhibiting abnormal activation of GLUT1 and GLUT4 [62]. The synergistic effects of GS-Rg3 on human ovarian cancer growth and angiogenesis when combined with cyclophosphamide were examined. Twenty-eight female glandless mice were selected for intraperitoneal injection of ginsenoside GS-Rg3 and CTX after inoculation with ovarian cancer cells, SKOV-3. The results showed that ginsenoside Rg3 alone or in combination with CTX significantly inhibited the growth and angiogenesis of ovarian cancer [65,66].

Another study revealed that Rg3 combined with radiotherapy effectively inhibited angiogenesis in CT-26 transplanted tumors (reducing CD31+ microvessel density) and prolonged the survival time of tumor-bearing mice [64].

Different concentrations of GS-Rh2 were transplanted into three prostate cancer cell lines (LNCaP, PC3 and DU145) in nude mice to measure the change in tumor volume over time.

### 2.3. Molecular Mechanisms Underlying Angiogenesis Inhibition

Another study showed that GS-Rg3 was able to target multiple signaling pathways induced by hypoxia, such as inhibiting the expression of HIF-1α, COX-2 and NF-κB and downregulating VEGF expression in human esophageal and renal cell carcinomas [67,68,69].

Inhibition of angiogenesis by ginsenosides is associated with a decrease in VEGF and MMP protein and expression. Ginsenoside Rg3 has been reported to reduce VEGF expression in acute leukemia patients through inactivation of PI3K/Akt and extracellular signal-regulated kinase ERK1/2 pathways [70]. It has been shown that Rg3 inhibits matrix MMP-9 activity and inhibits cyclooxygenase-2 (COX-2) expression in stimulated macrophages [71]. Therefore, the angiogenic biomarkers VEGF and MMP may be the main targets of ginsenoside Rg3 in inhibiting tumor angiogenesis.

GS-Rg3 inhibits the transforming growth factor (TGF)/Smad and extracellular signal-regulated kinase signaling pathways in human keloid fibroblasts (KFs). Administration of the drug significantly inhibited the proliferation, migration, invasion and angiogenesis of KF. In addition, in vivo results showed that ginsenoside Rg3 inhibited angiogenesis and collagen synthesis in KF [60].

Based on the mechanisms described above, the combination of Rg3 with anti-VEGF monoclonal antibodies or tyrosine kinase inhibitors may yield synergistic anti-angiogenic effects, which warrants further investigation [72].

This synergistic effect is attributed to the complementary mechanisms of action: Rg3 targets vascular endothelial cells to inhibit angiogenesis, while chemotherapeutic agents directly kill tumor cells. The combination thereby exerts effects on both tumor cells and the tumor microenvironment [73].

Based on the confirmed immunomodulatory activity of ginsenoside Rh2, researchers further explored its potential for synergistic antitumor effects when combined with immune checkpoint inhibitors. A study in 2023 found that the combination of Rh2 and anti-PD-L1 antibody could specifically activate the TBK1-IRF3 signaling pathway, significantly upregulate the expression of CXCL10 within tumors, and thereby promote the infiltration and functional activation of CD8^+^ T cells in tumors. Notably, when CD8^+^ T cells were specifically depleted, the antitumor effect of this combination therapy completely disappeared, directly confirming the core role of CD8^+^ T cells in this synergistic mechanism [74].

### 2.4. Clinical Evidence and Translational Potential

GS-Rg3 is one of the most studied ginsenosides and also one of the effective anticancer herbal ingredients. One of its anticancer properties is its anti-angiogenic effect. Since angiogenic factors control tumor growth and metastasis, angiogenic modulation of this process is critical for identifying new cancer treatment strategies.

Meta-analysis showed that GS-Rg3 in combination with chemotherapy improves short-term efficacy and overall survival, alleviates treatment adverse effects, reduces VEGF expression and increases CD4/CD8 T-cell ratio, and is a potential treatment strategy for non-small cell lung cancer (NSCLC) [75].

This study provided solid experimental evidence and a new strategy for Rh2 as an immunotherapy sensitizer. Additionally, another in vivo study showed that Rh2 alone had limited inhibitory effects on subcutaneous transplanted ovarian cancer tumors in nude mice; however, when combined with cisplatin, it not only significantly inhibited tumor growth and prolonged the survival of tumor-bearing mice, but also no superimposed toxicity was observed, suggesting that Rh2 has good safety and potential for enhancing efficacy in combination therapy [76].

## 3. Ginsenoside Regulates EMT in the TME of Cancer

**Figure 2:**
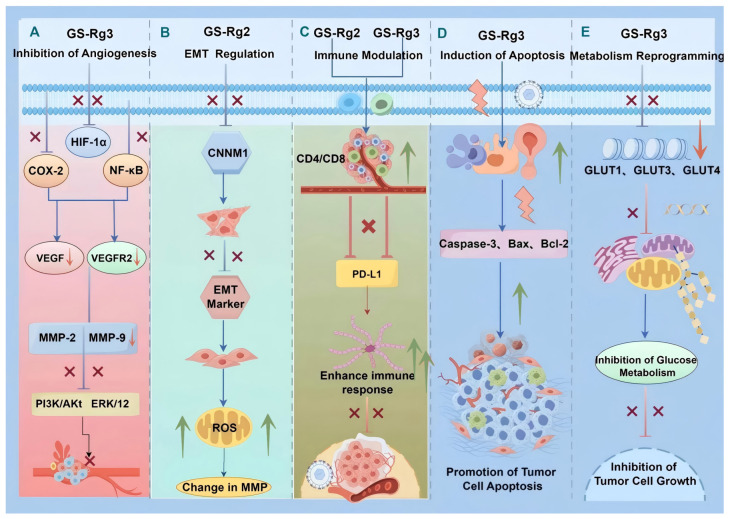
Anti-tumor mechanisms of ginsenosides: regulation of angiogenesis, EMT, immunity, apoptosis, and tumor metabolism. (**A**) GS-Rg3 inhibits angiogenesis by downregulating COX-2, HIF-1α, NF-κB, VEGF, VEGFR2, MMP-2/9, PI3K/Akt, and ERK1/2. (**B**) GS-Rg2 regulates EMT via modulation of CD4/CD8, CNNM1, EMT markers, and ROS. (**C**) Both GS-Rg2 and GS-Rg3 modulate immune responses by affecting CD4/CD8 and PD-L1. (**D**) GS-Rg3 induces apoptosis through caspase-3, Bax, and Bcl-2. (**E**) GS-Rg3 reprograms metabolism by inhibiting glucose metabolism. Created in BioRender. Li, W. (2026) https://BioRender.com/fp7lg75.

Numerous studies have found that epithelial–mesenchymal transition (EMT) is closely related to tumorigenesis. The diverse cytosolic material in the tumor microenvironment is associated with the development of EMT, which is manifested by the loss of epithelial markers (e.g., epithelial calmodulin, γ-linked protein) and the acquisition of bone marrow mesenchymal markers (e.g., fibronectin, wave proteins, neurocalmodulin). Ginsenosides regulate EMT and immunosuppression in tumor development (Table 2, Figure 2), as described below:

### 3.1. Ginsenoside Rb1 and Rg1: Modulating EMT Through Canonical Pathways

Ginsenoside Rb1 (GS-Rb1) has potent toxicity to tumor cells. It was found that EMT was an important player in GS-Rb1-mediated tumor suppression, while GS-Rb1 and its metabolic compound K were able to inhibit self-renewal in ovarian cancer patients and xenograft tumor models [92]. GS-Rb1 antagonizes hypoxia-induced E-cadherin downregulation and vimentin upregulation in SKOV3 and 3AO human ovarian cancer cells to reverse hypoxia-induced EMT, suggesting that ginsenoside Rb1 may be a potential drug candidate for the treatment of ovarian cancer [93]. GS-Rg1 was able to inhibit transforming growth factor β1 (TGF-β1)-induced invasion and migration of HepG2 hepatocellular carcinoma cells, and studies demonstrated that this inhibition was associated with the suppression of TGF-β1-induced EMT. When TGF-β1-induced HepG2 cells exhibited a mesenchymal phenotype, this was reversed after GS-Rg1 administration, and the cells exhibited a typical epithelial morphology, while GS-Rg1 also increased the expression of the epithelial phenotype marker E-cadherin and inhibited the expression of the mesenchymal phenotype marker vimentin. This study provides a rationale for GS-Rg1 as a potential inhibitor of invasive migration in human hepatocellular carcinoma [94,95].

### 3.2. Ginsenoside Rg3 and Its Stereoisomers: Dual Regulation of EMT

An interesting study was conducted to investigate whether GS-Rg3 inhibits EMT and lung cancer invasion through a glycobiological mechanism. Three types of non-small cell lung cancer cells, A549, H1299, and H358, were selected for the experiment. GS-Rg3 significantly altered EMT marker proteins, with elevated expression of E-cadherin and decreased expression of N-cadherin and vimentin, while FUT4 was downregulated together with shFUT4, and EMT was significantly inhibited. The results demonstrated that GS-Rg3 inhibited EMT by downregulating FUT4-mediated EGFR inactivation and blocking MAPK and NF-κB signaling pathways to inhibit EMT and lung cancer invasion. GS-Rg3 may be a potentially effective drug for the treatment of lung cancer [96]. Cisplatin is the first-line chemotherapeutic agent for lung cancer, but reduced sensitivity may limit its use. GS-Rg3 sensitizes hypoxic lung cancer cells to cisplatin by blocking NF-κB-mediated EMT and stem cell properties [97].

Of greater clinical significance, Rg3 has been shown to enhance the sensitivity of lung cancer cells to cisplatin. Studies have confirmed that under hypoxic conditions, Rg3 re-sensitizes drug-resistant lung cancer cells to cisplatin by blocking NF-κB-mediated epithelial–mesenchymal transition (EMT) and stemness properties [79]. This mechanism is further supported by evidence that Rg3 inhibits the Nrf2-mediated cytoprotective system, thereby enhancing cisplatin-induced cytotoxicity [98]. These findings provide a theoretical basis for the combination of Rg3 and cisplatin in the treatment of drug-resistant lung cancer.

20-Rg3 is a differential isomer of GS-Rg3, including 20(s)-Rg3 and 20(R)-Rg3, which has been shown to also inhibit EMT and thus lung cancer migration and invasion, with the difference that 20-Rg3 significantly suppresses the expression of the epithelial marker E-cadherin by inhibiting TGF-β1-induced EMT initiation. Glioblastoma multiforme (GBM) is one of the most malignant intracranial tumors in humans. 20(s)-Rg3 reverses temozolomide resistance by inhibiting the progression of epithelial–mesenchymal transition in glioblastoma [80].

This suggests that the combination of 20(S)-Rg3 and temozolomide may represent an effective therapeutic strategy for glioblastoma, particularly in cases involving EMT-associated drug resistance.

Cancer aggressiveness is one of the important factors affecting the poor prognosis of ovarian cancer patients, and EMT is an important mechanism mediating the invasive metastasis of cancer cells. Ginsenoside 20(S)-Rg3 targets HIF-1α to block hypoxia-induced epithelial–mesenchymal transition in ovarian cancer cells, and targeting the EMT process with a more effective and less toxic compound to inhibit metastasis has great therapeutic value in the treatment of prostate cancer [99]. 20(R)-Ginsenoside Rg3 affects colorectal cancer tumor stem cell properties and epithelial–mesenchymal transition through the SNAIL signaling axis [82].

Collectively, these findings demonstrate that ginsenoside Rg3 and its stereoisomers suppress EMT through convergent mechanisms involving NF-κB, HIF-1α, and TGF-β/SNAIL signaling, leading to upregulated E-cadherin and downregulated N-cadherin and vimentin expression.

### 3.3. Ginsenoside Rb2: Convergence of EMT and Immunosuppression

The expression of TGF-β1 was significantly higher in human colon cancer samples than in normal samples. Ginsenoside Rb2 (GS-Rb2) inhibited the growth, adhesion and metastasis of human colorectal cancer cells by suppressing the TGF-β/Smad signaling pathway to inhibit EMT. Western Blot analysis further confirmed that GS-Rb2 inhibited the expression of TGF-β1, Smad4, and phosphorylated Smad2/3 in vitro and in vivo [84].

Rb2 exhibits a dual regulatory effect on EMT and immunosuppression, targeting the TGF-β/Smad pathway while modulating MMP-2/9 expression to inhibit tumor invasion and metastasis.

The immunomodulatory effects of ginsenosides are mediated through a network of signaling pathways and cytokines. Suppression of NF-κB signaling by Rg3 reduces COX-2 expression and subsequent PGE2 production, thereby alleviating MDSC-mediated immunosuppression. Ginsenosides also regulate key cytokines in the tumor immune microenvironment: CK inhibits IL-1β, IL-6, and IL-17 secretion while reducing COX-2 and Arg-1 expression in MDSCs; Rg1, on the other hand, promotes dendritic cell activation through the production of IL-8 and IP-10. Additionally, matrix remodeling enzymes, including MMP-2 and MMP-9, which facilitate both angiogenesis and immunosuppression in the tumor microenvironment, are suppressed by ginsenosides such as Rg3. This multi-pathway regulatory pattern makes ginsenosides versatile modulators of the tumor immune microenvironment.

## 4. Ginsenoside Regulates Immunoreactive Cells in the TME of Cancer

It is well known that some cancer therapies can temporarily weaken the immune system, while enhancing the immune response may play an important role in cancer treatment [100]. Ginseng has a long history in improving the immunity of Asian patients. Ginseng saponins directly affect tumor cells by inducing apoptosis of cancer cells and inhibiting the growth of cancer cells and enhance antitumor immunity by activating cytotoxic T lymphocytes and natural killer cells [101,102]. More and more studies show that GS-Rh2 can improve immunity. One study reported that GS-Rh2 enhanced the antitumor immune response and enhanced the cytotoxicity of spleen lymphocytes by triggering the infiltration of CD4+ and CD8a+ T lymphocytes in B16-F10 melanoma cells from xenograft tumor tissue [85]. Another study found that GS-Rh2 can downregulate the expression of CASP1, INSL5 and OR52A1 in MCF-7 cells and upregulate the expression of CLINT1, ST3GAL4 and C1orf198, suggesting that GS-Rh2 can induce apparent methylation changes in genes involved in immune response and tumorigenesis, thereby enhancing immunogenicity and inhibiting cancer cell growth [103]. GS-Rh2 inhibits t-cell acute lymphoblastic leukemia (T-ALL) by blocking the PI3K/Akt/mTOR signal pathway and upregulates IL-2 and INF by downregulating IL-4, IL-6, IL-10, CD3 and CD45- γ; enhances the immunity in the spleen; and increases the number of natural killer cells [86].

Research has shown that the combination of Rh2 with an anti-PD-L1 antibody can enhance the efficacy of immunotherapy by increasing intratumoral CXCL10 levels and reprogramming the function of CD8^+^ T cells [74]. This finding provides a new theoretical basis for the application of Rh2 as a sensitizing agent in immunotherapy.

These studies indicate that GS-Rh2 has the ability to enhance immune response and may play a role in cancer prevention and treatment. Bone-marrow-derived suppressor cells (MDSCs) play an important role in tumor immune escape in the tumor microenvironment and are significantly increased in the peripheral blood of tumor patients or tumor mouse models. In order to identify epigenetic regulatory genes and elucidate the most significant signaling pathway for the effect of Rh2, Lee’s team conducted a genome-wide methylation analysis. The results showed that the epigenetic methylation changes in genes and cell-mediated immune response-related pathways were regulated by Rh2, including downregulation of CASP1, INSL5 and OR52A1, and upregulation of CLINT1, ST3GAL4 and C1orf198, thereby enhancing immunogenicity and inhibiting the growth of MCF-7 breast cancer cells [104]. The effects of GS-Rg3 on MDSC and the changes in tumor stem cell-like cells (CSCs) and EMT were evaluated by various methods. MDSC promotes the occurrence of breast cancer by strengthening the stem and promoting EMT. The anticancer effect of GS-Rg3 is due to its downregulation of MDSC, thereby inhibiting cancer growth and EMT in breast cancer. Therefore, we suggest that regulating MDSC through GS-Rg3 treatment is an effective treatment strategy for breast cancer patients [87].

Ginsenoside CK (GS-CK) has a significant effect on MDSCs in mouse models of colorectal cancer xenotransplantation.

As a final gut metabolite of ginsenosides, CK exhibits immunomodulatory activity that positions it as an ideal candidate for combination therapy. Studies have demonstrated that CK regulates the tumor microenvironment by suppressing tumor angiogenesis-related proteins and downregulating immunosuppressive cells, including myeloid-derived suppressor cells (MDSCs) [105,106]. This dual regulation of MDSCs and tumor-associated macrophages (TAMs) provides a strong rationale for exploring CK in combination with conventional chemotherapeutics such as 5-FU or oxaliplatin for colorectal cancer treatment.

The results showed that the apoptosis rate of early MDSCs in the GS CK treatment group was higher than that in the control group. At the same time, GS-CK can significantly reduce the expression of immunosuppression-related genes COX-2 and Arg-1 and inhibit IL-1β and the secretion of IL-6 and IL-17, thereby inhibiting the progress of mouse CT26 cancer. The effect of ginseng activity on macrophages extends to the immunosuppressive phenotype [74].

Tumor-associated macrophages (TAMs) are one of the myeloid immunosuppressive cells in the tumor microenvironment. More and more studies have shown that M2 macrophages are tumor-promoting macrophages associated with tumor progression and poor prognosis. They are responsible for releasing immunosuppressive cytokines, chemokines and growth factors, such as arginase, VEGF, platelet-derived growth factor and interleukin-10 (IL-10). They can reduce tumor-specific cytotoxic T lymphocyte reactions and promote tumor angiogenesis [107,108]. Ginsenosides can transform TAM into M1 macrophages and enhance the antitumor activity of M1 macrophages [109,110]. Zhu et al. confirmed that ginsenoside Rh2 can better reduce the expression levels of vascular endothelial growth factors MMP2 and MMP9 on TAM and transform TAM from the M2 type to the M1 type, thereby inhibiting tumors [111]. Lymphocytes are the key component of the adaptive immune system, which can directly combine with target cells and destroy or release cytokines to enhance immune effects and participate in immune responses. For example, GS-Rh2 enhances the antitumor immune response of a melanoma mouse model. In the melanoma mouse model, GS-Rh2 enhanced the infiltration of T lymphocytes in the tumor, and the immune response induced by GS-Rh2 can be transferred to other mice, inhibit the growth of melanoma, and improve the survival time of mice [112,113].

Dendritic cells (DCs), the most powerful professional antigen-presenting cells, participate in the interaction between innate immunity and adaptive immune response and play an important role in inhibiting tumor occurrence and development [114,115]. However, the inhibitory function of dendritic cells in tumor patients is an important reason for tumor escape from immune recognition and tumor progression. Both immature and mature DCs in a tumor-bearing host may inhibit T cell function through the tumor microenvironment. The functional activation or transformation of DC in tumor patients is an important mechanism of ginseng as an immunomodulator. Studies have shown that ginsenoside, as a functional component of ginseng, participates in enhancing the function of dendritic cells in the tumor microenvironment. Ginsenosides activate dendritic cells, promote adaptive immune response, and play an anticancer role in tumor-bearing mice. Huang et al. found that GS-Rg1 has an auxiliary effect on DC by promoting the production and secretion of cytokines and chemokines IL-8 and IP-10 and further explored its combined antitumor activity with OVA in lymphoma mouse models [90]. GS-Rh1 can stimulate DCs to promote T cell proliferation and enhance the antitumor activity of LPAK by treating PHA and IL-2 [116].

## 5. Ginsenosides Regulate Metabolic Reprogramming in the Tumor Microenvironment

Cancer cells undergo metabolic reprogramming within the tumor microenvironment (TME), characterized by altered glucose and lipid metabolic patterns [117]. This metabolic shift not only fuels the uncontrolled proliferation of cancer cells but also serves as a key prerequisite for their invasive and metastatic behavior. Ginsenosides have been shown to directly interfere with glucose and lipid metabolic reprogramming in cancer cells through targeted regulation of metabolism-related signaling pathways and protein molecules [118]. This mechanism represents an important anticancer pathway of ginsenosides and synergistically operates with other effects—including inhibition of tumor angiogenesis and modulation of epithelial–mesenchymal transition (EMT)—to enhance overall anticancer efficacy [119]. To systematically summarize the regulatory effects and molecular targets of ginsenosides on cancer glucose and lipid metabolic reprogramming, the key findings are detailed in Table 3:

### 5.1. Ginsenosides Inhibit Glucose Metabolism Reprogramming

Glucose metabolic reprogramming is a hallmark of cancer cells, which rapidly take up and utilize glucose by upregulating glucose transporter (GLUT) expression and enhancing glycolytic flux. Even under aerobic conditions, cancer cells preferentially rely on glycolysis for energy production—a pathway that rapidly generates ATP and provides biosynthetic precursors for nucleic acids, proteins, and other macromolecules [123]. Ginsenosides interfere with this process primarily by modulating GLUT family members and glycolysis-associated signaling pathways [124].

Ginsenoside Rg3 directly downregulates GLUT1, GLUT3, and GLUT4 protein expression in multiple cancer cell types [17]. In a rat model of gastric precancerous lesions, elevated GLUT1, GLUT3, and GLUT4 expression in lesional tissues was significantly reduced following intraperitoneal administration of ginsenoside Rg3, accompanied by decreased CD34+ microvessel density. Downregulation of GLUT expression directly limits glucose uptake by cancer cells, thereby blocking glucose metabolism at the initial step. Additionally, ginsenoside Rg3 indirectly suppresses VEGF and GLUT1 expression by inhibiting HIF-1α and NF-κB signaling [67,125], achieving concurrent inhibition of tumor angiogenesis and glucose uptake—a dual blockade of both blood supply and metabolic substrate availability.

Ginsenoside Rg3-based liposomes exploit GLUT1 overexpression on triple-negative breast cancer (TNBC) cells for tumor-targeted drug delivery [126]. By replacing cholesterol as a membrane component, Rg3 enables its glycosyl moiety to specifically bind GLUT1 on cancer cell surfaces. Docetaxel-loaded Rg3 liposomes are preferentially internalized by 4T1 TNBC cells and accumulate at tumor sites. Following cellular uptake, these liposomes further suppress key glycolytic enzyme activity and reduce glycolytic product accumulation [120]. This strategy not only enhances chemotherapeutic drug targeting but also leverages Rg3-mediated inhibition of glucose metabolic reprogramming for synergistic anticancer effects.

Ginsenoside Rg1 alters mitochondrial membrane potential in TNBC cells by inducing reactive oxygen species (ROS) generation [53]. Elevated ROS production impairs mitochondrial structure and function, thereby suppressing aerobic respiration. Disruption of mitochondrial membrane potential directly affects mitochondrial energy metabolism and reduces ATP production. This mechanism limits glucose utilization at the mitochondrial level, diminishing energy supply to cancer cells. Concurrently, Rg1-induced ROS activate apoptotic pathways, promoting cancer cell death alongside metabolic inhibition [53].

Ginsenoside Rh2 inhibits glucose metabolism in acute lymphoblastic leukemia cells by modulating the PI3K/Akt/mTOR signaling pathway—a central regulator of cellular metabolism and proliferation [127]. Rh2 blocks PI3K/Akt/mTOR pathway activation and downregulates downstream glycolysis-related enzyme expression [121]. This directly suppresses glycolytic flux and reduces glucose utilization. Simultaneously, inhibition of this pathway attenuates cancer-cell-proliferative capacity, achieving dual metabolic and proliferative suppression.

### 5.2. Ginsenosides Regulate Lipid Metabolism Reprogramming

Lipid metabolic reprogramming represents another key metabolic adaptation in cancer cells, which accumulate large quantities of lipids through enhanced de novo synthesis and uptake [128]. These lipids not only serve as energy sources but also participate in membrane synthesis, signaling molecule generation, and TME modulation [129]. Ginsenosides interfere with lipid metabolic reprogramming primarily by targeting cholesterol metabolism-related genes and signaling pathways, with squalene epoxidase (SQLE) emerging as a core action target [130].

20(S)-ginsenoside Rg3 specifically regulates SQLE expression in ovarian cancer cells [131]. SQLE, a rate-limiting enzyme in the de novo cholesterol synthesis pathway, is frequently overexpressed in ovarian cancer. 20(S)-Rg3 upregulates SQLE expression, thereby altering cholesterol metabolic flux. Disruption of cholesterol homeostasis affects cancer cell membrane structure and fluidity, reducing invasive and metastatic potential [83]. Furthermore, reversal of cholesterol metabolic reprogramming diminishes the release of lipid-associated signaling molecules into the TME, alleviating immunosuppressive effects [132].

20(R)-ginsenoside Rg3 modulates lipid metabolic reprogramming in colorectal cancer cells through the SNAIL signaling axis–a core pathway regulating both EMT and lipid synthesis. 20(R)-Rg3 inhibits SNAIL axis activation and downregulates downstream lipid synthesis-related protein expression [82]. This reduces de novo lipid synthesis and abnormal lipid accumulation in colorectal cancer cells. The concomitant reduction in lipid synthesis further suppresses EMT, achieving synergy between metabolic inhibition and metastasis suppression.

Ginsenoside Rb2 exerts inhibitory effects on lipid metabolism in colorectal cancer cells by suppressing the TGF-β/Smad signaling pathway [122]. This pathway enhances lipid synthesis and uptake through downstream transcription factor regulation. Rb2 significantly downregulates TGF-β1, Smad4, and phosphorylated Smad2/3 expression in both in vitro and in vivo settings. Inhibition of this pathway directly impairs lipid synthesis enzyme activity in colorectal cancer cells, reducing lipid production. Concurrently, this effect suppresses EMT and diminishes invasive and metastatic potential.

Ginsenoside compound K (CK) indirectly modulates lipid homeostasis within the TME by inhibiting lipid metabolism in tumor-associated macrophages (TAMs) [105]. TAMs undergo lipid metabolic reprogramming and promote cancer cell proliferation and metastasis through the release of lipid-related cytokines [133,134]. CK downregulates COX-2 and arginase-1 expression in TAMs, thereby inhibiting their lipid metabolic processes [135]. This reduces TAM secretion of cytokines such as IL-1β and IL-6 into the TME, alleviating immunosuppression and attenuating lipid-related signaling effects on cancer cell metabolism.

### 5.3. Synergistic Relationship Between Ginsenoside-Mediated Regulation of Metabolic Reprogramming and Other Anticancer Effects

Ginsenoside-mediated regulation of cancer cell metabolic reprogramming does not operate in isolation but synergizes with other anticancer mechanisms—including inhibition of tumor angiogenesis, modulation of EMT, and enhancement of antitumor immunity—to form a multi-targeted anticancer network. This synergistic interaction amplifies anticancer efficacy and reduces the likelihood of drug resistance development.

Ginsenoside Rg3 concurrently downregulates GLUT1 expression while inhibiting VEGF-mediated tumor angiogenesis [136]. Suppression of angiogenesis reduces tumor blood supply and impairs glucose delivery, while GLUT1 downregulation directly limits cancer cell glucose uptake. These combined effects block glucose availability at both systemic and cellular levels, achieving synergistic inhibition of glucose metabolism. Conversely, inhibition of glucose metabolism further impairs cancer cell VEGF secretion, creating a bidirectional regulatory loop that reinforces angiogenesis suppression [137].

Ginsenoside Rg3 improves the TME’s metabolic status by inhibiting cancer cell glucose metabolic reprogramming while modulating myeloid-derived suppressor cells (MDSCs) to enhance antitumor immunity [138]. MDSC downregulation alleviates TME immunosuppression and enhances cytotoxic T lymphocyte and natural killer cell activity. Simultaneously, inhibition of cancer cell glucose metabolism reduces accumulation of metabolic byproducts such as lactic acid within the TME. Reduced lactic acid levels ameliorate TME acidification, boost immune cell activity, and further potentiate antitumor immune responses.

## 6. Modulation of Tumor Microenvironment by Ginsenoside Nano-Drug Delivery System

Ginsenoside has a significant effect in treating tumors, but its poor water solubility leads to a slow oral dissolution rate and low bioavailability [11,139].

The solubility of ginsenosides varies with structure: PPD-types such as Rg3, Rh2, and CK possess fewer sugar chains and are therefore lipophilic and nearly water-insoluble, whereas PPT-types like Rg1, with more sugar moieties, exhibit better water solubility. This structural difference dictates solvent selection and influences in vivo behavior. Following oral administration, most ginsenosides remain stable in gastric fluids, with retention rates exceeding 90%, yet display poor permeability across Caco-2 cell monolayers (<1 × 10^−6^ cm/s), indicating that limited intestinal absorption is a major barrier to bioavailability [140]. Once absorbed, they circulate in the bloodstream bound to serum proteins, though some are susceptible to esterase-mediated hydrolysis. Hepatic metabolism adds further complexity: multi-sugar PPD-type ginsenosides such as Rb1 and Rb2 require deglycosylation by gut microbiota to generate absorbable metabolites like compound K [141], whereas ginsenoside Rg3 primarily modulates phase II metabolic enzymes, including UDP-glucuronosyltransferases and Glutathione S-transferases. This metabolic conversion both restricts bioavailability and yields the active metabolite compound K [142]. Ultimately, excretion occurs via the kidneys or bile. Thus, the stability and metabolic fate of ginsenosides vary considerably across different biological environments—absorption is limited in the gut, transport is relatively stable in circulation, and transformation occurs primarily in the liver. These pharmacokinetic characteristics pose significant challenges for clinical application.

Therefore, the development of ginsenoside preparations with good stability, high bioavailability and targeted drug delivery is of great significance for clinical treatment. Liposomes are nanoscale vesicles formed by encapsulating drugs with a lipid-like bilayer, mainly composed of polar phospholipids as membrane materials and added cholesterol [143,144]. Liposomes can be adsorbed on the outer wall of target cells because their functional structure is similar to biofilm so that drugs can act on target sites. In cancer treatment, cancer-associated fibroblasts (CAFs) can affect drug penetrability and immunosuppressive TME. Liposomes carrying ginsenosides combine the advantages of both and have an improving effect on cancer treatment (Table 4), as described below:

A study on targeted treatment of triple-negative breast cancer (TNBC) with GS-Rg3 liposomes found that GS-Rg3 can replace cholesterol to make liposomes have the activity of targeting GLUT1 overexpressed in TNBC tumor cells. This finding improved the limited therapeutic effect of docetaxel (DTX) on TNBC encapsulated in traditional cholesterol liposomes. GS-Rg3 liposome (Rg3lp/DTX) was loaded with DTX. Rg3 LP/DTX could be preferentially taken up by 4T1 cells and accumulated in tumor sites through the interaction between the glycosyl part of Rg3 exposed on the surface of the liposome and the overexpression of glucose transporter 1 (GLUT1) on tumor cells. This study provides a treatment method with a simple preparation process and good therapeutic effect for the treatment of TNBC [145]. Paclitaxel (PTX) is a natural anticancer drug, which has been widely used in the treatment of breast cancer, ovarian cancer, some head and neck cancers and lung cancer. But resistance to PTX is a key challenge of cancer chemotherapy. Although various targeted drug delivery systems, including nanoparticles and liposomes, are effective in the treatment of multidrug-resistant cancer, their efficacy is limited by the immunosuppressive TME. Zhu et al. established functional Rg3-based PTX liposomes for the treatment of PTX resistance in breast cancer based on the physicochemical properties and strong antitumor activity of GS-Rg3. Rg3-PTX-LPs can be specifically distributed in MCF7/T cancer cells and the TME at the same time, mainly through the recognition of GLUT-1. Compared with conventional cholesterol liposomes, Rg3 PTX LPs have significantly improved drug resistance reversal ability and antitumor effects in vivo. The TME-remodeling mechanism of Rg3 PTX LPs includes inhibiting the activation of the IL-6/STAT3/p-STAT3 pathway, repolarizing the original M2 macrophage into an antitumor M1 phenotype, inhibiting MDSCs, reducing CAFs and collagen fibers in the TME, and promoting tumor cell apoptosis [111]. In the treatment of brain tumors, the shortcomings of cholesterol also greatly limit the application of traditional liposomes [148]. The GS Rg3 liposome system (Rg3 LPs), compared with cholesterol liposomes (C-LPs), not only significantly improved the absorption and penetration of glioma cells in vitro but also significantly enhanced the targeting activity and intratumor diffusion of glioma cells in vivo. Rg3 PTX LPs can significantly prolong the median survival time of c6 mice/rats by activating the immune microenvironment in glioma, promoting T cell immune response, increasing the number of CD8+ T cells, increasing the ratio of M1/M2, and reducing regulatory T cells and myelogenous inhibitory cells. This study proves that GS Rg3 is a good cholesterol substitute in the liposome administered and has a synergistic effect with the loaded anticancer drugs [146]. Hong et al. have developed a multifunctional liposome system (Rh2 lipo) based on GS-Rh2. Cholesterol and polyethylene glycol are replaced by Rh2, and Rh2 is also used as a membrane stabilizer, a long cycle stealth agent, an active target ligand, and a chemotherapy adjuvant. In the xenograft model of 4T1 breast cancer, GS-Rh2 loaded with paclitaxel achieves efficient tumor growth inhibition, and Rh2-lipo can reshape the tissue structure of the TME and reverse the immunosuppressive environment. This discovery provides another innovative potential system with multiple functions for anticancer drug delivery [149]. When ginsenoside liposomes are used as tumor-targeting drugs, ginsenoside can not only be used as an adjuvant to chemotherapy but also as functional membrane material. Ginsenoside liposomes provide a new platform for the delivery of anticancer drugs and may lead to a new era of cancer nanocarrier therapy. Ginsenoside combined with PTX has a synergistic inhibitory effect on the proliferation of human gastric cancer cells. In one study, a new multifunctional liposome system was developed. Ginsenoside was used as an adjuvant to chemotherapy and a membrane stabilizer. These liposomes have a long blood circulation time and positive targeting ability, thus creating multiple functions of liposomes and promoting drug delivery of gastric cancer cells [150].

Recent advances in smart drug delivery systems have further expanded the therapeutic potential of ginsenosides. Leveraging the amphiphilic nature of ginsenoside Rg3 itself, researchers have developed self-assembled nanoparticles that function as “carrier-free” delivery systems. For instance, Rg3 co-assembles with flavonoids like naringenin via non-covalent interactions to form stable nanoparticles (∼100 nm), enabling nose-to-brain delivery that bypasses the blood–brain barrier for Alzheimer’s disease intervention. Stimuli-responsive nanoplatforms have also been engineered to achieve tumor microenvironment-triggered drug release. Examples include hyaluronic acid-modified hollow MnO_2_ nanoparticles loaded with Rg3, which exhibit pH-responsive release in acidic tumor environments with drug loading capacity up to 42.42%, significantly enhancing antitumor efficacy against triple-negative breast cancer. These next-generation formulations transform ginsenosides from passive payloads to active components of intelligent delivery systems.

## 7. Conclusions and Perspectives

Ginsenosides have been shown to have significant anticancer effects and multi-level, multi-target effects [151]. Ginsenosides can exhibit antitumor ability by inhibiting tumor cell growth, inducing tumor cell cycle arrest, promoting tumor cell death, inhibiting tumor cell invasion and metastasis, regulating tumor cell autophagy and enhancing immune regulation. At the same time, ginsenosides can act synergistically with various antitumor drugs to increase the sensitivity of tumor cells to chemotherapy and reduce adverse effects, which has great potential for clinical application [152]. However, there is a certain understanding of ginsenosides in antitumor, but more in-depth studies are still needed to provide an experimental basis for ginsenosides and their metabolites in the treatment of tumors and to lay the foundation for clinical treatment.

Equally important is the safety profile of ginsenosides. Accumulating evidence demonstrates their selective cytotoxicity against cancer cells while sparing normal cells. Ginsenoside Rh3 exhibits a selectivity index of 3.04 between human colon cancer HCT116 cells and normal colonic epithelial cells [153]; ginsenoside Rg3 induces DNA damage and apoptosis in osteosarcoma cells while protecting normal fibroblasts from chemically induced injury [154]. Animal toxicity studies reveal no significant adverse effects following maximum-dose administration of total ginsenosides via inhalation or oral routes. Clinical trials further confirm their favorable safety profile in healthy volunteers, patients with hepatic dysfunction, and high-intensity exercise populations, with adverse event rates comparable to placebo [155]. This “high efficacy, low toxicity” characteristic positions ginsenosides as promising candidates for long-term adjuvant therapy and combination regimens with conventional chemotherapeutics.

This favorable safety profile, combined with recent advances in related technologies, is accelerating the clinical translation of ginsenosides. Synthetic biology has enabled the biosynthesis of rare ginsenosides Rh2 and F1 in tobacco plants, and the complete gene cluster for compound K biosynthesis in Panax species has been elucidated [156,157]. Monoclonal antibody technologies now extend beyond analytical tools to production applications, including one-step immunoaffinity purification of ginsenoside Rb1 and genetic strategies that increase secondary metabolite yields by over two-fold [158]. Clinically, ginsenoside Rg3 eye drops have completed phase III trials, and Rg3 injections are undergoing phase II evaluation, with indications expanding to ophthalmic and neurodegenerative diseases. Together with ongoing advances in delivery systems, these developments position ginsenosides for an expanding role in comprehensive cancer therapies.

In this paper, we review the research progress of ginsenoside modulation of the TME to regulate tumors; a deeper understanding of the impact of the TME on cancer development can provide new targets, ideas and approaches for cancer therapy. Targeted TME is a promising anticancer therapy strategy, and the development of drugs injected with targeted TME also has broad prospects as future anticancer drug therapy. Although the target of ginseng’s anticancer effects is unclear, current studies have demonstrated that ginseng’s active ingredients can influence tumor–TME interactions. However, the bioavailability of ginsenosides is limited. In the future, the delivery system can be used to deliver ginsenosides to the effective site to increase the curative effect, which makes the research of traditional Chinese medicine reach a new height. The summary study in this paper will help to find more ginsenosides that can inhibit tumor cells by improving the tumor microenvironment and provide more ideas for the development of cancer therapeutic drugs.

## Figures and Tables

**Table 1:** Detailed information on ginsenosides targeting the TME to regulate cancer.

Ginsenoside	Cancer	Target Makers	Related Hallmark	Reference
Rg3	Colorectal cancer	Ki-67/ ANGPT1/ CCL13 decrease	Tumor angiogenesis	[54]
Rg3	Colorectal cancer	VEGF/ COX-2/NF-κB decrease	Tumor angiogenesis	[55]
Rg3	Liver cancer	VEGF/Bcl-2 decrease Bax increase	Tumor angiogenesis	[56]
Rg3	Human esophageal cancer/renal cell carcinoma	VEGF/ HIF-1α/COX-2 decrease	Tumor angiogenesis	[57]
Rg3	Thyroid cancer	VEGF-A/ MMP-2 decrease	Tumor angiogenesis	[51]
Rg3	Acute leukemia	VEGF/PI3K/ Akt decrease	Tumor angiogenesis	[58]
Rg3	NSCLC	VEGF/CD4/CD8 decrease	Tumor angiogenesis	[59]
Rg3	KF	TGF/Smad decrease	Tumor angiogenesis	[60]
Rg3	Advanced metastatic melanoma	VEGF/MMP-9/ P53 decrease	Tumor angiogenesis	[61]
Rg3	Hepatocellular carcinoma	CD105 decrease	Tumor angiogenesis	[55]
Rg3	Gastric cancer	VEGF/ GLUT1/3/4 decrease	Tumor angiogenesis	[62]
Rh2	Prostatic cancer	CD31/VEGF/ PDGF/CNNM1 decrease	Tumor angiogenesis	[63]
Rg1	Triple-negative breast cancer	VEGF/MMP-2 decrease	Tumor angiogenesis	[53]

**Table 2:** Detailed information on ginsenoside-mediated regulation of EMT in the TME of cancer.

Ginsenoside	Cancer	Target Makers	Related Hallmark	Experimental Model	Reference
Rb1	Ovarian cancer	Wnt/β-catenin decrease	EMT	In vitro (ovarian cancer cells); In vivo (ovarian cancer xenografts)	[77]
Rg1	Liver cancer	Smad7 increase	EMT	In vitro (HepG2 cells)	[78]
Rg3	Lung cancer	mTOR/HIF-1α/VEGF decrease	EMT	In vitro (A549/H1299/H358 cells)	[68]
Rg3	Lung cancer	NF-κB decrease	EMT	In vitro (hypoxic lung cancer cells); In vivo (lung cancer xenografts)	[79]
20(S)-Rg3	Glioblastoma multiforme	Wnt/β-catenin decrease	EMT	In vitro (GBM cells); In vivo (GBM xenografts)	[80]
20(S)-Rg3	Ovarian cancer	DNMT3A decrease	EMT	In vitro (ovarian cancer cells)	[81]
20(R)-Rg3	Colorectal cancer	SNAIL/PI3K/Akt decrease	EMT	In vitro (colorectal cancer cells); In vivo (colorectal cancer xenografts)	[82]
20(S)-Rg3	Ovarian cancer	SQLE increase	EMT	In vitro (ovarian cancer cells)	[83]
Rb2	Colorectal cancer	TGF-β1/Smad4/ Smad2/3 decrease	EMT	In vitro (colorectal cancer cells); In vivo (colorectal cancer xenografts)	[84]
Rh2	Melanoma	Src/STAT3 decrease	EMT	In vitro (B16-F10 cells); In vivo (melanoma xenografts)	[85]
Rh2	Pancreatic Cancer	CARD9/BCL10/ MALT1/NF-κB increase	Immunosuppression	In vitro (pancreatic cancer cells)	[85]
Rh2	Acute lymphoblastic leukemia	PI3K/Akt/mTOR decrease	Immunosuppression	In vitro (leukemia cells); In vivo (leukemia model mice)	[86]
Rg3	Breast cancer	GLUT1/ALDH1/ OCT4/KLF4 decrease	Immunosuppression	In vitro (breast cancer cells); In vivo (breast cancer xenografts)	[87]
Rh2	Lung cancer	MMP-2/MMP-9 decrease	Immunosuppression	In vitro (lung cancer cells); In vivo (lung cancer xenografts)	[88]
CK	Colorectal cancer	PI3K/Akt/CD44/CD133 decrease	Immunosuppression	In vivo (colorectal cancer xenografts)	[89]
Rg1	Lymphoma	IL-8/IP-10 increase	Immunosuppression	In vitro (lymphoma cells); In vivo (lymphoma model mice)	[90]
Rh1	Cancer	IL-2 increase	Immunosuppression	In vitro (immune cell lines)	[91]

**Table 3:** Detailed information on ginsenoside-mediated regulation of glucose and lipid metabolic reprogramming in cancer.

Ginsenoside	Cancer	Target Makers	Related Hallmark	Experimental Model	Reference
Rg3	Gastric cancer	GLUT1/3/4/ CD34 decrease	Glucose metabolism	In vivo (rat model)	[62]
Rg3	Breast cancer	GLUT1/ GLUT4 decrease	Glucose metabolism	In vivo (breast cancer xenografts)	[87]
Rg3	Triple-negative breast cancer	GLUT1 decrease	Glucose metabolism	In vitro (4T1 cells); In vivo (tumor-bearing mice)	[120]
20(S)-Rg3	Ovarian cancer	SQLE increase	Lipid metabolism	In vitro (ovarian cancer cells); In vivo (ovarian cancer xenograft)	[83]
20(R)-Rg3	Colorectal cancer	SNAIL decrease	Lipid metabolism	In vitro (colorectal cancer cells); In vivo (colorectal cancer xenograft)	[82]
Rh2	Acute lymphoblastic leukemia	PI3K/Akt/ mTOR decrease	Glucose metabolism	In vitro (leukemia cells); In vivo (leukemia model)	[121]
Rb2	Colorectal cancer	TGF-β1/ Smad4/ Smad2/3 decrease	Lipid metabolism	In vitro/In vivo	[122]

**Table 4:** Detailed information on ginsenoside nanoparticle drug carriers targeting the TME of cancer.

Ginsenoside	Cancer	Target Makers	Nanoparticle Drug Carrier	Experimental Model	Reference
Rg3	Triple-negative breast cancer	GLUT1 decrease	Lipidosome	4T1 cell-derived xenograft in BALB/c mice	[145]
Rg3	Breast cancer	IL-6/ STAT3/ p-STAT3 decrease	Lipidosome	MCF-7/T cell-derived xenograft	[111]
Rg3	Glioma	CD8+ decrease	Lipidosome	C6 orthotopic glioma model in rats	[146]
Rg3	Gastric cancer	GLUT1 decrease	Lipidosome	SGC-7901 xenograft in nude mice	[147]
Rg5	Gastric cancer	GLUT2 decrease	Lipidosome	SGC-7901 xenograft in nude mice	[147]
Rh2	Gastric cancer	GLUT5 decrease	Lipidosome	SGC-7901 xenograft in nude mice	[147]

## Data Availability

No new data were created or analyzed in this study. Data sharing is not applicable to this article.

## Abbreviations

The following abbreviations are used in this manuscript:

TME	Tumor microenvironment
GS-Rg3	Ginsenoside Rg3
RTK	Receptor tyrosine kinase
TAE	Transcatheter arterial embolization
PTC	Papillary thyroid carcinoma
MMP-2	Matrix metalloproteinase 2
VEGF	Vascular endothelial growth factor
COX-2	Cyclooxygenase-2
NSCLC	Non-small cell lung cancer
KF	Keloid fibroblasts
TGF	Transforming growth factor
GS-Rh2	Ginsenoside Rh2
GS-Rg1	Ginsenoside Rg1
ROS	Reactive oxygen species
MMP	Mitochondrial membrane potential
EMT	Epithelial–mesenchymal transition
GS-Rb2	Ginsenoside Rb2
T-ALL	T-cell acute lymphoblastic leukemia
MDSCs	Marrow-derived suppressor cells
CSCs	Stem cell-like cells
GS-CK	Ginsenoside CK
TAMs	Tumor-associated macrophages
IL-10	Interleukin-10
DCs	Dendritic cells
CAFs	Cancer-associated fibroblasts
TNBC	Triple-negative breast cancer
GLUT1	Glucose transporter
PTX	Paclitaxel
C-LPs	Cholesterol liposomes
